# Successful management of catheter injury or refractory infection by partial replantation of peritoneal dialysis catheters: a retrospective observational study

**DOI:** 10.1186/s12882-024-03847-w

**Published:** 2025-02-03

**Authors:** Seyoung Ryou, Eun Jeong Ko, Hoon Suk Park, Byung Ha Chung, Cheol Whee Park, Chul Woo Yang, Yong-Soo Kim, Hyung Duk Kim, Yaeni Kim

**Affiliations:** 1 Division of Nephrology, Department of Internal Medicine, College of Medicine, The Catholic University of Korea, Seoul, Republic of Korea; 2 Dr. Yong-Soo Kim’s Internal Medicine Clinic and Hemodialysis Unit, Seoul, Republic of Korea; 3 Division of Nephrology, Department of Internal Medicine, College of Medicine, Eunpyeong St. Mary’s Hospital, The Catholic University of Korea, 1021 Tongil-ro, Eunpyeong-gu, Seoul, 03312 Korea; 4 Division of Nephrology, Department of Internal Medicine, College of Medicine, Seoul St. Mary’s Hospital, The Catholic University of Korea, 222 Banpo-daero, Seocho-gu, Seoul, 06591 Korea; 5 Division of Nephrology, Bucheon St. Mary’s Hospital, 327 Sosa-ro, Wonmi-gu, Bucheon, 14647 Bucheon, Republic of Korea

**Keywords:** Exit site infection, Peritoneal dialysis, Replantation, Mechanical catheter damage

## Abstract

**Background:**

The revised 2023 guidelines from the International Society for Peritoneal Dialysis (ISPD) emphasize salvage methods for treating refractory catheter-related infections, or mechanical catheter damage. This approach preserves the existing catheter by manipulating only the outer cuff above the peritoneum, avoiding hemodialysis transfer. We investigated the effectiveness of the partial replantation technique.

**Methods:**

In this retrospective single-center study (January 2021 - December 2023), outcomes for nine patients undergoing salvage methods were compared with 58 patients receiving de novo catheter insertion. We assessed exit-site infection (ESI), tunnel infection (TI), peritonitis, and catheter dysfunction. The salvage method entailed distal cutting of the impaired catheter and attaching a new segment using a connector with a PD adaptor and transfer set.

**Results:**

Nine patients (four males, mean age 56 years, average PD duration 66 months) employed the salvage method. Post-procedure, one patient (11.1%) reported ESI, one (11.1%) experienced TI, three (33.3%) developed peritonitis, and two (22.2%) required catheter removal. No procedural complications or catheter dysfunctions were observed. In the control group, ESI occurred in six patients (10.3%), TI in one (1.7%), peritonitis in 11 (19.0%), catheter removal in seven (12.1%), and catheter dysfunction in one (1.7%). Kaplan-Meier analysis showed no statistical difference between the groups: ESI (*p* = 0.306), TI (*p* = 0.094), peritonitis (*p* = 0.838), catheter dysfunction (*p* = 0.694), and catheter removal (*p* = 0.393).

**Conclusions:**

This study supports the non-inferiority and effectiveness of the salvage method compared to de novo insertion in managing ESI or TI and mechanical catheter damage.

**Supplementary Information:**

The online version contains supplementary material available at 10.1186/s12882-024-03847-w.

## Background

Peritoneal dialysis (PD) has served as a vital treatment option for patients with end-stage kidney disease (ESKD), as well as for older patients [1], with continuous improvements aimed at reducing complications since its introduction in 1975 [2]. In Korea, approximately 5,610 patients underwent PD, accounting for 4.4% of ESKD patients [3]. Catheter-related infections, specifically exit site infections (ESI) and tunnel infections (TI), significantly burden patients by causing technical setbacks and leading to the discontinuation of PD [4]. When PD catheter damage occurs, particularly near the proximal portion of the exit site, the removal of the old catheter and simultaneous insertion of a new one is often required.

In 2023, the International Society for Peritoneal Dialysis (ISPD) [5] revised guidelines on catheter-related infections. They endorsed salvage methods such as cuff shaving and exit site relocation within the surgical intervention section for refractory cases, assigning them the same recommendation grade. The strategy of simultaneous removal and reinsertion is frequently adopted for intractable cases. Nevertheless, this approach necessitates interrupting peritoneal dialysis and transitioning to hemodialysis due to peritoneal manipulation. Moreover, manipulations for new peritoneal catheter insertion may lead to complications such as hemorrhage, peritoneal leakage, catheter malposition, and omental trapping. Additionally, transitioning to hemodialysis, even temporarily, may be essential, necessitating the disruption of PD.

When considering a shift to hemodialysis, salvage methods provide the benefit of preserving the peritoneum, enabling the immediate resumption of PD after the procedure. Several studies have documented their efficacy and clinical outcomes over multiple years across various countries. Nevertheless, the studies reported so far are mostly descriptive in nature, with limited case series that lack control groups. The scarcity of cases does not suffice to establish statistical significance. In this context, we highlight the effectiveness and non-inferiority of partial replantation as one of the salvage methods, compared to a control group with de novo catheter insertion.

## Methods

### Patient enrollment

A retrospective review was undertaken involving nine patients who experienced either catheter mechanical damage or catheter-related infections between January 2021 and December 2023. This study focused on comparing individuals who underwent the salvage method or the partial replantation technique, with an emphasis on the exit relocation procedure, to 58 patients who received de novo catheter insertions during the same timeframe. Since nine patients received partial replantation, those undergoing de novo catheter insertions during this interval were selected for comparison. This study was conducted in compliance with the Declaration of Helsinki. The study protocol received approval from the Institutional Review Board (IRB) of Seoul St. Mary Hospital (approval number: KC24RASI0343). Due to the retrospective nature of this study, the IRB waived the requirement for informed written consent from participants.

### Definition of catheter-related infection and damage

Exit site infection is defined as the presence of purulent discharge, with or without erythema at the catheter-epidermal interface. Tunnel infection is identified by erythema, swelling, tenderness, or induration along the catheter tunnel, occasionally accompanied by fluid collection, as detected through ultrasound [5]. These catheter-related infections are confirmed through positive culture results. Different types of mechanical damage to catheters include perforation, tear, and rupture. Perforation is characterized by a small hole in the catheter that lead to fluid leakage. A tear represents a significant type of damage, yet it does not completely sever the catheter. Catheter rupture is defined as a complete severance of the catheter. Partial replantation was carried out on patients who had intact inner cuffs and exhibited no signs of infection beneath the peritoneum. Importantly, peritonitis should be excluded via physical examination, including assessment for abdominal pain, tenderness and the clarity of PD fluid, along with PD fluid analysis and laboratory results that indicate infection or inflammation.

### The salvage procedure

**Fig. 1:**
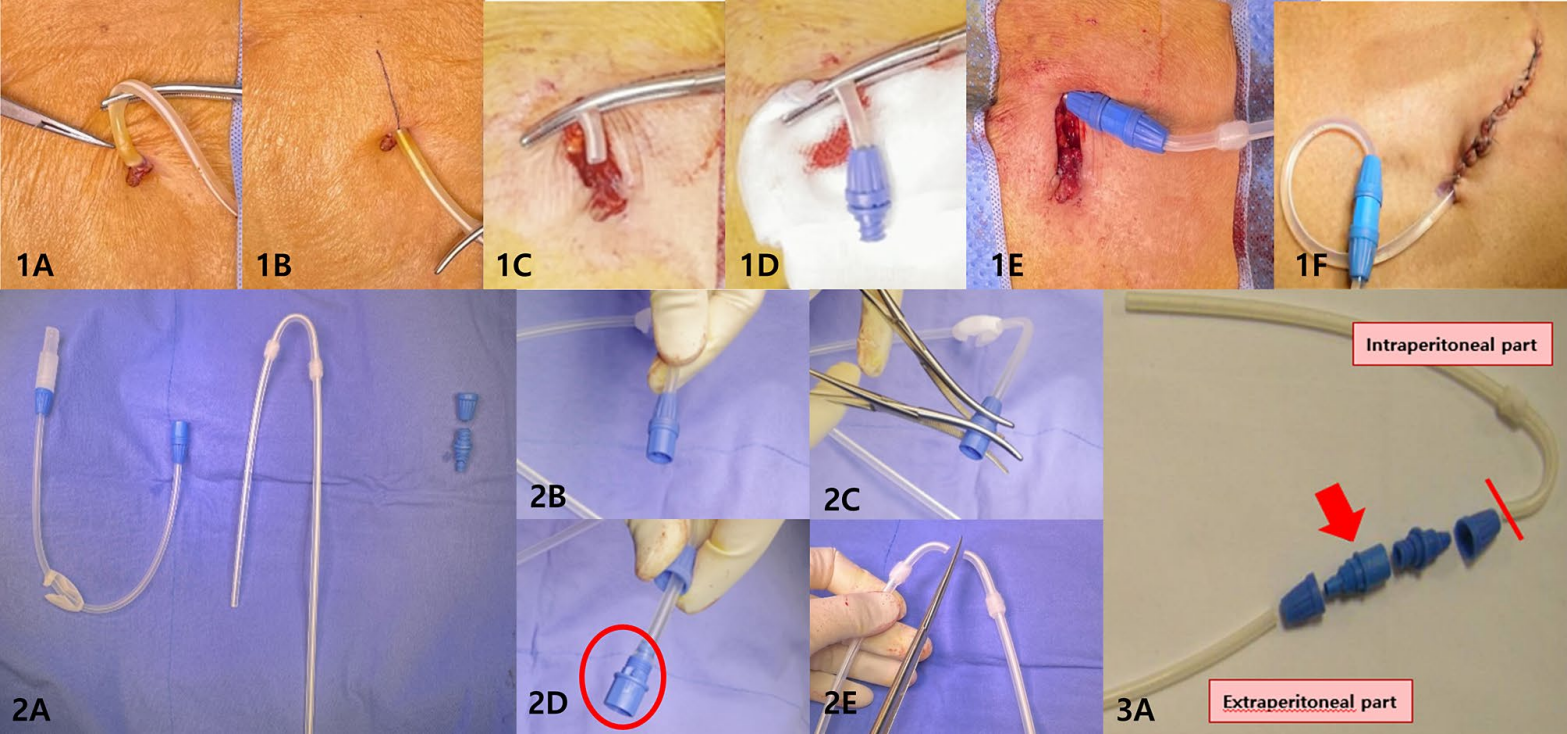
Surgical procedure illustrating the partial replantation technique. Make an incision at the PD catheter exit and cut the proximal part of the catheter (**1B-C**). Use Tenckhoff catheter, transfer set, and PD catheter adaptor (**2A**). Disassemble the transfer set (red circle) with Kelly forceps (**2B-D**), Cut the new catheter to the desired length between cuffs (**2E**). The connector device is created using a piece of the transfer set (red arrow) and two PD adaptors (**3A**). The intraperitoneal part consists of the old catheter covered by skin, while extraperitoneal part is the newly cut catheter. Attach Luer lock adaptor to the end of the old catheter (**1D**), connect it to the new catheter (**1E**) using the connector device, and create a new tunnel (**1F**)

The partial replantation procedure involved administering local anesthesia with 1% lidocaine. Initially, a longitudinal incision was made in the skin and subcutaneous tissues along the catheter from the exit site, followed by dissection to expose the outer cuff and the tunnel. Subsequently, the catheter was severed proximal to the site of rupture or infection, encompassing the outer cuff. Two PD adaptors and a piece of transfer set (Stay Safe catheter extension Luer Lock 25 cm; Fresenius Medical Care Deutschland GmbH, Bad Homburg, Germany) were utilized as connectors to link the end of the remaining old catheter to the extra-peritoneal segment of the new catheter (Catheter Adaptor Luer Lock; Fresenius Medical Care Deutschland GmbH). After connecting, the skin incision was elongated downwards from the original exit site to insert the connector into the tunnel, ensuring a 1 cm gap between the exit site and the end of the distal PD adaptor. After confirming the catheter function using saline irrigation, the wound was closed in layers **[**Fig. 1**]**.

### Statistical analysis

All continuous variables are presented as mean ± standard deviation. All categorical variables are presented as the number of patients and proportions. They were compared using Fisher’s exact test. The clinical outcomes- ESI, TI, peritonitis and PD catheter survival after catheter revision- were evaluated by Kaplan-Meier survival analysis and log-rank testing. Statistical significance was established at *p* < 0.05. All statistical analyses were conducted with GraphPad Prism version 10.1.0 (GraphPad Software, San Diego, CA, USA) and SPSS version 24 (IBM Corp., Armonk, NY, USA).

## Results

### Characteristics of patients undergoing catheter replantation versus de novo catheter insertion

A total of nine patients underwent the PD catheter partial replantation procedure, with a mean age of 56 years. Among them, four were male, accounting for 44.4% of the total. The total dialysis period for these patients was 594 patient-months, with a mean individual dialysis period of 66 months. All patients were treated for hypertension and among them, three patients (33.3%) had diabetes, four patients (44.4%) had coronary artery disease, and two patients (22.2%) had heart failure. Additionally, three patients (33.3%) had undergone kidney transplantation. Baseline characteristics according to patient groups are presented in Table 1. The reasons for catheter replantation included mechanical damage in seven patients, ESI in one patient, and TI in another. The causative microorganisms isolated were *Corynebacterium amycolatum* in one case and *Corynebacterium striatum* in the other. Before planning the surgical intervention, an appropriate antibiotics therapy regimen was administered, including at least 3 weeks of vancomycin to ensure adequate treatment duration and target identified microorganisms, *Corynebacterium amycolatum* and *Corynebacterium striatum* (Table 2). One gram of intravenous cefazolin was administered as prophylaxis for both the salvage and de novo insertion groups. Additionally, one gram of intraperitoneal cefazolin was administered after the procedures.

**Table 1:** Patients baseline characteristics

	Partial replantation (*n* = 9)	De novo (*n* = 58)	*p*-value*
Age, year	55.56 ± 14.65	56.74 ± 12.89	0.847
Sex (n, %)			0.500
Men	4 (44.4%)	33 (56.9%)	
Women	5 (55.6%)	25 (43.1%)	
**Primary Renal Disease** (n,%)			0.058
Hypertension	1 (11.1%)	1 (1.7%)	
Diabetes	2 (22.2%)	15 (25.9%)	
CGN	6 (66.7%)	21 (36.2%)	
SLE	0 (0.0%)	2 (3.4%)	
ADPKD	0 (0.0%)	1 (1.7%)	
Unknown/other	0 (0.0%)	18 (31.0%)	
**Comorbidity**			
Hypertension	9 (100%)	44 (75.9%)	0.186
Diabetes	3 (33.3%)	23 (39.7%)	1.000
CAD	4 (44.4%)	9 (15.5%)	0.063
HF	2 (22.2%)	11 (19.0%)	1.000
CVA	2 (22.2%)	9 (15.5%)	0.635
Liver disease	1 (11.1%)	4 (6.9%)	0.526
COPD	0 (0.0%)	4 (6.9%)	1.000
Cancer	0 (0.0%)	7 (12.1%)	0.581
Autoimmune disease	0 (0.0%)	2 (3.4%)	1.000
IST medication	3 (33.3%)	7 (12.1%)	0.125

CGN; chronic glomerulonephritis, SLE; systemic lupus erythematosus, ADPKD: autodominant polycystic kidney disease, CAD; coronary artery disease, HF; heart failure, CVA; cerebrovascular accident, COPD; chronic obstructive pulmonary disease, IST; immunosuppressive therapy

*Mann-Whitney test; Fisher’s exact test

**Table 2:** Causes of Catheter partial replantation and their clinical courses

Case	Age/sex	PD vintage	Cause of replantation/ organism (if infection)	ESI/organism	Tunnel infection/organism	Peritonitis/organism	Removal
1	36/M	98 months	Catheter perforation	36 months/Corynebacterium species	-	36 months/ MRCNS (S. epidermidis)	
2	37/F	96 months	Catheter perforation		-	10 days/ Candida parapsilosis	46 days
3	49/F	96 months	Catheter perforation		-	23 months / no growth	
4	68/M	14 months	Tunnel infection/Corynebacterium Striatum		-	-	
5	45/M	85 months	ESI / Corynebacterium amycolatum		4 months / Corynebacterium Striatum	-	6 months
6	79/F	76 months	Catheter rupture			-	
7	60/F	32 months	Catheter tear			-	
8	63/M	1 month	Catheter tear			-	
9	63/F	96 month	Catheter tear			-	

PD; peritoneal dialysis, ESI; exit site infection

### Clinical outcomes of catheter-related infections, dysfunction, and peritonitis after catheter replantation versus de novo catheter insertion

**Fig. 2:**
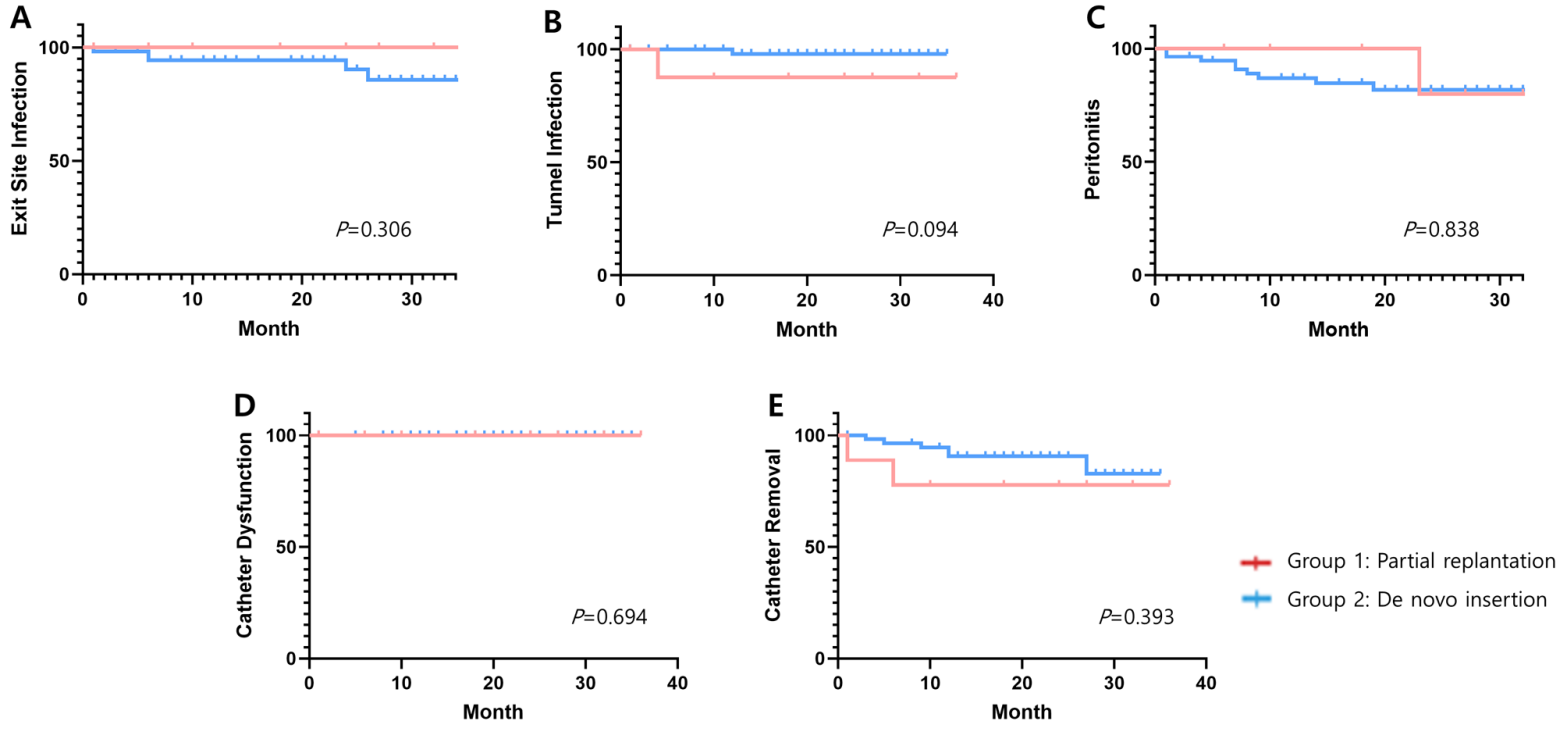
Kaplan-Meier analysis comparing clinical outcomes between partial replantation and de novo insertion of catheters. (**A**) Exit site infection (*p*: 0.306) (**B**) Tunnel infection (*p*: 0.094), (**C**) Peritonitis (*p*: 0.838) (**D**) Catheter dysfunction (*p*: 0.694), and (**E**) Catheter removal (*p*: 0.393), probabilities over time for both groups; Group1-Partial replantation, Group2-De novo insertion

After the replantation procedure, one patient experienced ESI (11.1%), and another developed TI (11.1%), while three patients (33.3%) developed peritonitis. The onset of infections occurred at 36 months for ESI, 4 months for TI, and 23 months for peritonitis. Eventually, two patients (22.2%) required PD catheter removal due to refractory infection and transitioned modality to hemodialysis. There were no instances of catheter dysfunction or procedure-related complications after partial replantation. The average follow-up period was 21.1 ± 9.8 months. In the de novo catheter insertion group, which served as the control group, the incidence rates were as follows: ESI occurred in six patients (10.3%), TI in one patient (1.7%), peritonitis in 11 patients (19.0%). Additionally, seven patients (12.1%) needed catheter removal due to refractory peritonitis, and one patient (1.7%) experienced catheter dysfunction. The follow-up duration was 20.6 ± 9.5 months. Regarding infection rates, ESI was 0.005(1/190) patient-years, TI was 0.005(1/190) patient-years and peritonitis was 0.011(2/190) patient-years in partial replantation group. In comparison, the de novo catheter insertion group exhibited ESI at 0.005(6/1192) patient-years, TI at 0.001(1/1192) patient-years, and peritonitis at 0.009(11/1192) patient-years. We employed Kaplan-Meier analysis and log-rank testing to assess the incidence of ESI, TI, peritonitis, catheter dysfunction, and catheter removal. The results indicated no significant differences in these clinical outcomes between the catheter replantation and control groups, with *p*-values of 0.306 for ESI, 0.094 for TI, 0.838 for peritonitis, 0.694 for catheter dysfunction, and 0.393 for catheter removal **[**Fig. 2**]**. Additionally, we categorized infections after the procedure into early and late infections with 1 month as the standard. There was no statistically significant difference between the two groups, with *p*-value of 0.487 for early infections and 0.869 for late infections [Supplemental Fig. 1]. In addition, there were no hypersensitivity reactions to the PD connector or catheter leaks during the follow-up period.

## Discussion

According to the 2023 ISPD revised guidelines [5], the initial treatment of catheter-related infections involves adequate antibiotic administration. However, if the infection persists, particularly in cases of refractory ESI or TI, salvage methods such as cuff saving and exit site relocation could be prioritized over the simultaneous removal and reinsertion technique. These approaches preserve the peritoneum, unlike the latter technique which entails peritoneal manipulation.

In cases of intractable infection where both the catheter and cuff are invaded by pathogenic organisms, complete catheter removal is often necessary to ensure eradication, as failure to eliminate the pathogens may result in persistent bacterial colonization. If the infection does not involve the inner cuff, considering partial replantation as a salvage method is feasible [5]. This approach requires several conditions, including a still-functioning inner conduit, a negative culture of dialysate, no signs of infection over the internal cuff area, and specifically, the presence of a near-cuff perforation.

Particularly, eschewing peritoneal manipulation offers numerous benefits. A study in the United States reported that up to 15.5% of PD-naïve patients required a follow-up procedure within 90 days post-initial PD catheter insertion due to complications such as bleeding, catheter obstruction, malposition, kinking, and peritoneal leakage [6]. Notably, the replantation method allows for the prompt resumption of PD without requiring a hemodialysis catheter (0 day vs. 16.6 days, *p* < 0.001). The use of a hemodialysis catheter, even in short-term, can potentially induce vessel damage, and prolonged dwelling can exacerbate this damage [7–9]. Additionally, partial replantation can be executed in an outpatient setting, which obviates the need for hospitalization thus potentially offering greater convenience and cost effectiveness. At our hospital, patients undergoing simultaneous catheter replacement typically required at least three nights and four days of hospitalization to monitor procedure-related complications.

Previous studies have shown that partial replantation is comparable to complete catheter replacement [10–13]. Our study corroborates earlier research, revealing that partial replantation was not inferior. In contrast to other studies, we opted for a control group comprising patients who had a de novo catheter insertion for initiating PD. Given the lengthy average PD vintage of 66 months in the intervention group, comparing these patients to those newly starting PD could potentially influence the clinical outcomes adversely. Nonetheless, despite this disparity in groups, which includes control patients who had never previously undergone peritoneal manipulation, there was no significant difference in clinical outcomes. This finding emphasizes the non-inferiority of the salvage method compared to de novo catheter insertion. Despite the potential for more complications and reduced durability from retaining the previous catheter below the connector, especially given their average 66-month duration of PD, our study found no inferiority in clinical outcomes. Furthermore, there was no significant difference in the infection rates between the catheter replantation and de novo groups.

We constructed the connector using two PD adaptors and a segment of a transfer set, which were cost-effective and easily obtainable since they are routinely stocked in the PD unit. Previous studies typically employed medical adhesive glue or a specific endoluminal connector to join the remaining portion of the old catheter with a new one [10, 12–14]. The endoluminal tube, either silicone-based or a titanium extender, was integrated into the inner lumen of both catheters, and the interfaces were sealed using type A glue or sutures. However, it is crucial to acknowledge that adhesive materials may trigger chemical reactions with certain types of catheters or surrounding tissues, and the tied method used to connect the catheters can result in dialysate leakage [14, 15]. The biological safety of using adaptors and transfer segments as connectors need further validation. Our long-term follow-up results suggest the relative biocompatibility of the connector device and propose that our approach may offer advantages in terms of reducing complications, enhancing availability, and improving cost-effectiveness.

Our study has some limitations, primarily its small sample size. The reasons for partial replantation were divided into two main categories: refractory catheter-related infection and mechanical catheter damage, encompassing a total of nine patients. Ideally, the clinical outcomes for each group would be compared separately using other techniques, such as simultaneous removal and reinsertion. However, this technique is less commonly performed compared to simultaneous removal and reinsertion and requires specific conditions, which limited the number of eligible cases. Therefore, we chose to combine these two groups into a single cohort for comparison with the de novo insertion group, rather than analyzing infection and mechanical damage cases separately or comparing each subgroup with other methods. Regardless of the reason for performing partial replantation, our findings suggest that its efficacy is comparable to de novo insertion, supporting partial replantation as a viable alternative.

Although the clinical outcomes, specifically infection rates, showed no significant difference between de novo catheter insertion and partial replantation, the proportion of infections appeared relatively high. This may, however, be attributed to our study’s small sample size. Notably, the peritonitis rate at our hospital was 0.242 episodes per patient-year, reflecting effective infection control and education measures. Therefore, the observed higher infection rate is more likely related to the limited sample size rather than an actual deficiency in infection control practices.

In our study, two patients eventually required their PD catheters removed. One patient underwent partial replantation due to catheter perforation, but developed refractory peritonitis with *Candida parapsilosis* cultured in the peritoneal fluid, leading to the cessation of PD and catheter removal 10 days after the procedure. While it is plausible that the infection was surgery-related, the patient’s history of multiple catheter-related infections and a diabetic foot ulcer suggest that hygiene issues may also have played a role. The other patient underwent partial replantation for refractory ESI with *Corynebacterium species*, but developed TI with *Corynebacterium* four months later. Despite two months of antibiotic treatment, the PD catheter was ultimately removed to eradicate the infection. Remarkably, this patient had a history of a fourth ESI and one episode of peritonitis with *Corynebacterium* species before the replantation. Although there were no signs of peritonitis with an identified pathogen at the time of replantation, colonization by *Corynebacteria* cannot be excluded.

Despite these limitations, our study provides valuable insights into the management of catheter-related complications using partial replantation. This minimally invasive method may offer a feasible alternative to conventional techniques, potentially reducing hospitalization time and preserving peritoneal integrity. Further large-scale studies are needed to validate these findings and explore the full potential of partial replantation in clinical practice.

## Conclusions

The partial replantation offers several advantages, including being less invasive, suitable for outpatient settings, and reducing the need for hemodialysis transfer by minimizing peritoneal irritation. Therefore, clinicians might consider this salvage method for patients experiencing either ESI or TI without peritoneal infection, as well as those with mechanical catheter damage near the proximal part of the cuff.

## Electronic supplementary material

Below is the link to the electronic supplementary material.

Supplementary Material 1

Supplementary Material 2

## Data Availability

All data generated or analyzed during this study are provided in the supplementary information files, which are available in Excel format.

## Abbreviations

ESI: Exit site infection
ESKD: End-stage kidney disease
ISPD: International Society for Peritoneal Dialysis
PD: Peritoneal dialysis
TI: Tunnel infection
