# Supplementary material for: Successful management of catheter injury or refractory infection by partial replantation of peritoneal dialysis catheters: a retrospective observational study
Source: BMC Nephrol. 2025 Feb 3;26:52. doi: 10.1186/s12882-024-03847-w (PMC11792682; doi:10.1186/s12882-024-03847-w)
Supplement: Supplementary file 2 — Supplementary Material 2 [file 12882_2024_3847_MOESM2_ESM.docx]

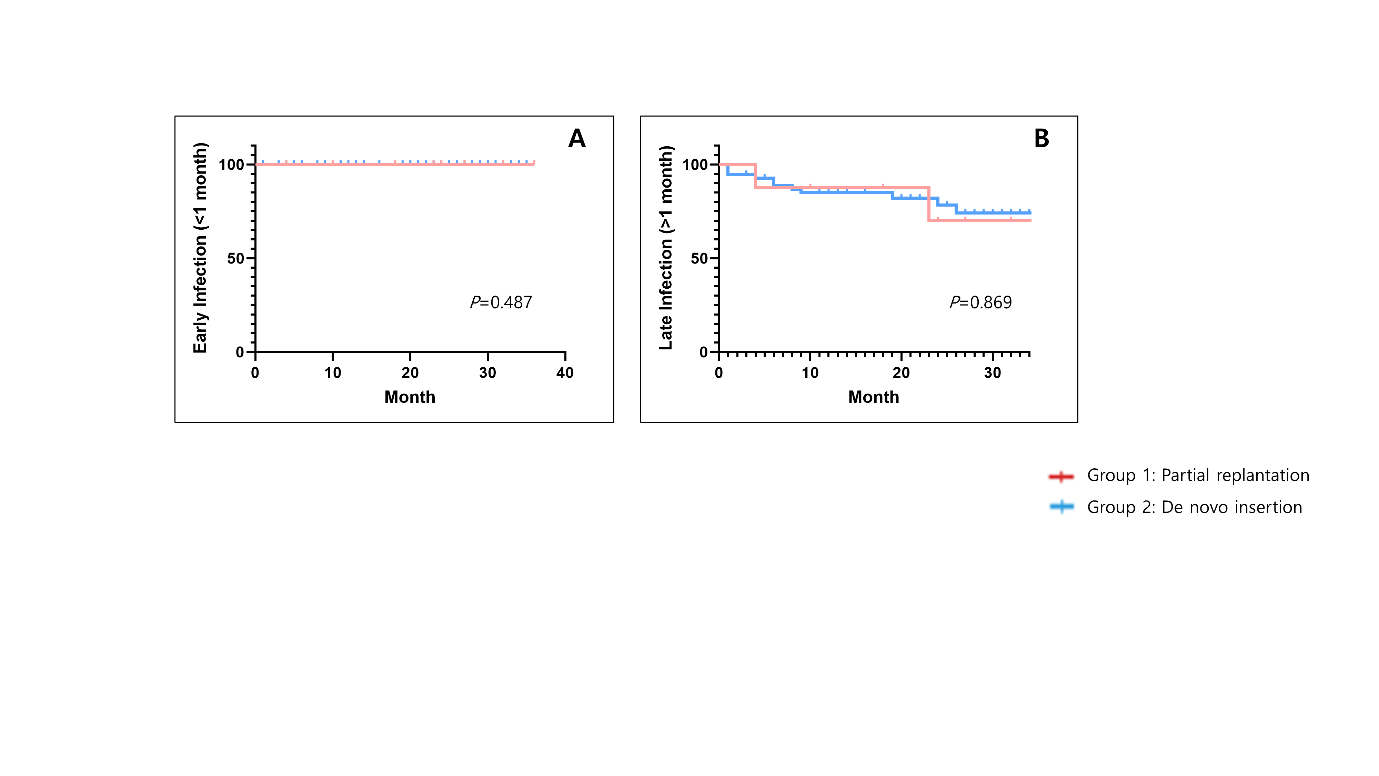


Supplemental Figure 1. Kaplan-Meier analysis comparing early and late infections between partial replantation and de novo catheter insertion. (A) Early infection (<1 month) (*p*: 0.487) (B) Late infection (>1 months) (*p*: 0.869). Probabilities over time for both groups; Group1-Partial replantation, Group2-De novo insertion.
